# Persistent Genital Arousal Disorder After Motor Vehicle Accident: A Case Report

**DOI:** 10.1089/whr.2020.0043

**Published:** 2020-09-09

**Authors:** Anamaria Parus, Meena Kanhai, John M. Tramont

**Affiliations:** University of Central Florida College of Medicine, Orlando, Florida, USA.

**Keywords:** gynecology, persistent genital arousal disorder, women's health

## Abstract

Persistent genital arousal disorder (PGAD) is a clinical syndrome characterized by persistent unwanted feelings of sexual arousal that are not associated with any specific sexual arousal or stimulus. The severity of symptoms range from mild to severe distress that interrupts daily life for patients. We present a 44-year-old previously healthy woman who developed PGAD after involvement in a motor vehicle accident in 2018. After sustaining lower spinal trauma, 3 months later, she began to experience intermittent tingling feelings in her clitoris. She noticed that exacerbations in back pain were also associated with PGAD symptoms. These symptoms progressively worsened to which she was constantly feeling as if she was on the verge of an orgasm. Her quality of life was severely diminished for 3 months, after which she presented to gynecology. Treatment of lidocaine patches applied to the sacrum were found to completely eliminate the feelings of clitoral stimulation. She also began physical therapy for the residual back pain. One year after initiation of treatment, she has experienced significant improvement in both the back pain and PGAD symptoms. Her quality of life is much improved and plans on continuing a treatment plan of lidocaine patches and physical therapy. Recognition of PGAD in women is important for clinicians as that it can go undiagnosed for long periods of time and can interfere with quality of life for patients.

## Introduction

Persistent genital arousal disorder (PGAD) is a clinical syndrome that is characterized by persistent unwanted feelings of sexual arousal that are not associated with any objective peripheral evidence of sexual arousal or stimulus.^1^ This condition was first identified and described by Leiblum and Nathan in 2001. The five diagnostic criteria for PGAD are as follows: the genital arousal feels intrusive and undesired, episodes persist for hours to days, episodes are triggered by various stimuli not limited to sexual stimuli or are spontaneous, orgasm does not necessarily result in resolution of symptoms, and there is at least a moderate feeling of distress about the condition.^2^

Although some women who experience mild and intermittent symptoms (non-PGAD) reportedly seem to embrace it, women with moderate or severe distress experience intense and continuous sensations, which are associated with feelings of shame, guilt, and anxiety.^3^ The condition can go undiagnosed for long periods of time, as patients might feel ashamed or think it is normal to experience the undesired genital arousal. Given the nature of this condition, incidence and prevalence have been difficult to elucidate.

Risk factors that have been associated with PGAD include restless leg syndrome and overactive bladder symptoms. A clinical study done by Waldinger and Schweitzer showed that 67% of the 18 women with a confirmed diagnosis of PGAD experienced restless legs concurrently with PGAD.^4^ Similar to PGAD, the symptoms of restless leg syndrome have a tendency to worsen while sitting and improvement occurs with movement or stimulation of the lower extremities.^4^ The study also showed that the majority of these women had symptoms of overactive bladder, such as urinary urgency and voiding frequency.^4^

Given these associations, it is reasonable to suspect (at least partly) a change of setpoint in neurophysiology provoked by undetermined alterations.^4^ Magnetic resonance imaging (MRI) studies of the women in this study showed evidence of mild to moderate pelvic varices in 55% of the cases.^4^

In addition, Leiblum and Goldmeier described five women who have developed PGAD either after withdrawing from selective serotonin reuptake inhibitor antidepressants or while using them.^5^ Another study found Tarlov cysts on MRI of 12 of the 18 patients (66.7%) who presented with PGAD symptoms.^6^ These findings are further evidence that the etiology can be complex and multifaceted.

Most women will have already identified simple interventions to alleviate symptoms, such as avoidance of tight clothing and prolonged sitting, but they have not proved useful at eradicating the symptoms.^7^ Treatment options that have proven to alleviate symptoms include benzodiazepines, amitriptyline, nortriptyline, gabapentin, venlafaxine, lidocaine gel, pudendal nerve block, and subcutaneous leuprolide.^8–10^

In this report, we review the case of a 44-year-old woman who developed symptoms of PGAD after sustaining spinal trauma after motor vehicle crash. Consent for this case report was obtained from the patient.

## Case Presentation

A 44-year-old previously healthy woman with a body mass index of 33.5 sustained a motor vehicle accident in March 2018. Patient's vehicle was rear-ended on the driver's side, in a diagonal manner. She was restrained with a seatbelt. She sustained lower back injury and reported experiencing numbness and tingling in the legs, as well as headaches and muscle spasms.

MRI of the lumbar spine done at that time showed multilevel disk pathology, including disk herniation at L2/L3, L3/4, L4/L5, and L5/S1. Additional findings at the L5/S1 level also demonstrated a radial tear and impingement on bilateral lateral recesses. There was no fracture noted. Approximately 3 months later, she started experiencing an intermittent tingling feeling in her clitoris, which progressively worsened to constantly feeling like she was on the verge of orgasm. Orgasm did not provide any relief of her symptoms and at times the patient felt like it was worsening them. Of note, the patient had not been on antidepressant medication before or during the onset of undesired genital arousal.

When asked about her quality of life, the patient stated, “It was terrible, you can't focus on anything, you can't wear any clothes from the waist down to try not to put any pressure at all in that area.” Driving, sitting in church, studying at her desk, and any other kind of pressure or vibrations in the genital area exacerbated her symptoms.

After 3 months of undesired genital arousal and feeling ashamed, “dirty and perverted,” she expressed her concerns to her medical team and was subsequently referred to the gynecology team. MRI of the pelvis 6 months after the injury showed disk pathology within the visualized lumbar spine (consistent with initial post-trauma MRI), no Tarlov cysts or bony fractures were identified. There was no radiological evidence of peripheral pudendal nerve compression. Physical examination revealed no abnormalities but was limited by her fear of response.

After careful review of her symptoms, medications, and contributing factors, a trial of lidocaine 4% patches, measuring 10 × 14 cm, applied to the sacrum was initiated. The patient was instructed to place the lidocaine patch centrally, right above the buttock crease.

She subsequently stated that the patches provided significant relief of symptoms. At her 3-month follow-up visit, she reported that, while wearing the patch, she experienced full resolution of the clitoral tingling sensation. She also reported the emergence of a new tingling sensation in the vagina. She was prescribed lidocaine gel, which initially provided limited relief. Overall, she reports great improvement in her quality of life since the onset of treatment with lidocaine patches. Although response to therapy has been adequate, she would prefer a more long-term alternative treatment over having to constantly reapply the lidocaine patch. She was presented with the various options to include pudendal nerve block and various medications but elected to continue with lidocaine as needed.

She returned to the office a year after the motor vehicle accident. At that visit, the patient was still experiencing residual back pain and reported that PGAD symptoms appeared to be linked to exacerbations in her back pain. She denied urinary urgency, urinary frequency, bladder or pelvic pain, or symptoms of restless leg syndrome. She started attending physical therapy for back pain and has noticed improvement with her back pain and associated PGAD symptoms. She reported being able to sleep through the night now, without having to wake up due to the genital arousal.

At 18 months follow-up, patient reported continued improvement but incomplete resolution. She experiences about one episode per week, which is always associated with increased lower back pain. She uses lidocaine patches during these episodes, which continue to provide relief. As of her last visit, she plans to continue using lidocaine and focus on improving back pain.

## Discussion

The most plausible explanation for the continuous arousal in PGAD is nerve entrapment.^11^ The entrapment could be central and be related to Tarlov cyst or herniated intervertebral disk, or it could be peripheral.^11^ If the source of the pathology is peripheral, surgical intervention could be curative.^11^ Innervation to the clitoris is provided by the dorsal branch of the pudendal nerve.^12^ In a study done by Klifto and Dellon on patients with PGAD, the most common location of nerve entrapment observed in their cohort of 34 patients was distal to the exit of the canal of Alcock, where the dorsal branch of the pudendal nerve exits the inferior pubic ramus canal, located where the inferior pubic ramus joins the pubic symphysis, and not at the exit of the canal of Alcock.^12^

In their experience, MRI studies have not been helpful in documenting the site of compression.^12^ In the case of our patient, it is likely that the nerve entrapment is due to the herniated intervertebral disks, but peripheral entrapment cannot be ruled out. The patient's lumbar MRI demonstrated multilevel disk pathology, including disk herniation and impingement on the lateral recesses. Although there is no radiological evidence of sacral pathology, given the lumbar MRI findings, it is likely that there is also pathology at S1/S2. If her PGAD is caused by herniated lumbar disks, nerve blocks, ablation, and possibly diskectomy could be beneficial. If her entrapment is peripheral, she would likely benefit from bilateral neurolysis of the dorsal pudendal nerve. Further evaluation will be offered to look for permanent solutions.

The lidocaine patches work by targeting free nerve endings in the dermis or mucosa, thereby producing temporary loss of sensation in a limited area.^13^ Nerve impulse conduction is blocked by lidocaine due to sodium channel blockade.^13^ The higher the concentration of anesthetic in the patch, the higher the rate of penetration.^13^ Transdermal delivery of anesthetic has shown to have relatively little penetration, with maximal depth of 5 mm, but since our patient is experiencing relief of symptoms, we suspect the lidocaine is penetrating just deep enough to affect the nerve roots.^13^ The patient applies the patches centrally, just above the buttock crease, likely affecting the S1 and/or S2 roots and likely having an effect on the pudendal nerve.

As previously described in literature, PGAD can be a psychologically distressing condition that severely impacts quality of life and can be very demoralizing for the patient. Although our patient did not seek counseling, it would seem appropriate to offer counseling by someone trained in sexual medicine.

This case illustrates the importance of exploring genital symptoms in patients with spinal or pelvic trauma and postdelivery. In this study, the patient's PGAD symptoms initially presented after moderate spinal column trauma. Another case report noted that the patient's PGAD presumably arose from pelvic trauma, after she sustained a large perineal hematoma associated with horseback riding incident.^10^ Therefore, asking patients who have sustained trauma to these regions about associated symptoms of PGAD may result in faster diagnosis and quicker treatment.

Considering that overactive bladder is a prevalent urogenital complaint, it would be beneficial for urologists and urogynecologists to start asking these patients if they are experiencing symptoms of PGAD as well.

Owing to the nature of the symptoms, patients are often reluctant to express their concerns to clinicians. The nonjudgmental assessment of sexual function, arousal, libido, and satisfaction with sexuality is essential for making a diagnosis of PGAD.^14^ Showing empathy, developing a trustful relationship, and promoting conversation can help patients feel more comfortable discussing their symptoms, which may improve timeliness of diagnosis and implementation of treatment. Greater public awareness through the use of trustful medical websites could also help women seek care faster.

Although more evidence has emerged in literature about this condition, there is still a need for research in finding the most effective way to diagnose and provide effective treatment to women suffering with PGAD.

## Abbreviations Used

MRI: magnetic resonance imaging
PGAD: persistent genital arousal disorder
